# An Atypical Presentation of Childhood Paraganglioma with Seizures: A Case Report and Review of the Literature

**DOI:** 10.1155/2023/6637802

**Published:** 2023-11-27

**Authors:** Elizabeth Eberechi Oyenusi, Uzoamaka Felicia Nwigbo, Oluwadamilola Moromoke Oladipo, Blessing Ebele Kene-Udemezue, Kasarachi Pauline Akowundu, Khadijah Omobusola Oleolo-Ayodeji, Oluwaseun Adunni Afoke, Funmilayo Oluwatoyin Babatunde, Felix Makinde Alakaloko, Gabriel Kolawole Asiyanbi, Ezekiel Olayiwola Ogunleye, Abiola Olufunmilayo Oduwole, Foluso Ebun Afolabi Lesi

**Affiliations:** ^1^Endocrinology and Metabolism Unit, Department of Paediatrics, College of Medicine, University of Lagos, Lagos University Teaching Hospital, Lagos, Nigeria; ^2^Department of Paediatrics, Lagos University Teaching Hospital, Lagos, Nigeria; ^3^Department of Radiodiagnosis, Lagos University Teaching Hospital, Lagos, Nigeria; ^4^Paediatric Surgery Unit, Department of Surgery, Lagos University Teaching Hospital, Lagos, Nigeria; ^5^Department of Anaesthesia, College of Medicine, University of Lagos, Lagos University Teaching Hospital, Lagos, Nigeria; ^6^Cardiothoracic Unit, Department of Surgery, College of Medicine, University of Lagos, Lagos University Teaching Hospital, Lagos, Nigeria; ^7^Neurology Unit, Department of Paediatrics, College of Medicine, University of Lagos, Lagos University Teaching Hospital, Lagos, Nigeria

## Abstract

**Introduction:**

A paraganglioma (PGL) is a tumour derived from extra-adrenal chromaffin cells of the sympathetic paravertebral ganglia of the thorax, abdomen, and pelvis. Cardiovascular manifestations predominate but neurological symptoms like seizures can occur requiring a high index of suspicion for prompt diagnosis and treatment. *Case Description*. A 14-year-old girl was referred to the paediatric neurology unit for recurrent headaches of one-year duration, vomiting of 2 months duration, and an episode of generalized tonic-clonic seizures, 2 weeks prior to presentation. There was an associated history of impaired vision, palpitations, diaphoresis, and easy fatigability. Her blood pressure ranged from 150/101 to 160/120 mmHg. The brain CT scan was normal. ECG showed left ventricular hypertrophy. Abdominal USS revealed a right para-aortic mass necessitating 24-hour urine normetanephrine which was markedly elevated–1695.34 mcg/24 h (100–500). An abdominal CT scan confirmed a paraganglioma in the right para-aortic region. A multidisciplinary team consisting of paediatric endocrinologists, radiologists, anaesthetists, paediatric and cardiothoracic surgeons, and the intensive care unit (ICU) team was involved in the peri and postoperative management of the child. Intraoperative challenges were hypertension and hypotension (following tumour excision). She was nursed in the ICU for 48 hours. Histology results confirmed paraganglioma. Postoperative urine normetanephrines done a month after surgery had reverted to normal. Her blood pressure has remained normal 6 months after surgery, and no other symptoms have recurred.

**Conclusion:**

In evaluating aetiology of childhood hypertension, endocrine causes must be considered though they are rare. The occurrence of paraganglioma is uncommon and can present in unusual ways such as seizures. Measurement of blood pressure in children is advocated as part of routine health care. Clinicians must explore the aetiology of seizures and not merely control them with anticonvulsant therapy.

## 1. Introduction

Neuroendocrine tumors can arise from neural crest-derived cells or paraganglia. Functional tumours are also called chromaffin tumours because they produce catecholamines which yield a brown-black colour (chromaffin) on oxidation after staining with chromium slats [1]. These tumors commonly arise from adrenal medulla and are known as pheochromocytoma (PCC) while a paraganglioma (PGL) is a tumour derived from extra-adrenal chromaffin cells of the sympathetic paravertebral ganglia of the thorax, abdomen, and pelvis [2, 3].

Abdominal PGLs occur near the renal vessels or the organ of Zuckerkandl, which is localized around the origin of the inferior mesenteric artery and is the largest extra-adrenal collection of chromaffin tissue [1]. Paragangliomas also arise from parasympathetic ganglia located along the glossopharyngeal and vagal nerves in the neck and at the base of the skull but usually do not produce catecholamines. Paragangliomas constitute 15–20% of chromaffin-cell-related tumours [1–4].

Secondary hypertension is commoner in children with PCCs and PGLs accounting for just 0.5–2% out of the 11% attributed to endocrine causes [2]. In addition to the rarity of PGLs generally, seizures are an unusual presentation occurring in 5.4% (5 out of 93) of a large series [5]. Furthermore, it is reported that many doctors will hardly consider PGLs when exploring possible causes of seizures. However, undiagnosed secretory PGLs can lead to complications from severe hypertension and other cardiovascular complications. Indeed, some cases have only been found at autopsy [6]. This case is reported to highlight the unusual first presentation of a child with PGL on account of seizures, thereby presenting first, to the paediatric neurologist.

## 2. Case Presentation

A 14-year-old female adolescent was referred to the paediatric neurology unit on account of recurrent headaches of one-year duration, projectile vomiting of 2 months duration, and an episode of generalized tonic-clonic seizures. Headache was sudden in onset, localized to the frontal region, throbbing in nature, nonradiating, not worse at any time of the day, aggravated by noise/stress, pain score of 7/10, relieved temporarily with analgesic (paracetamol) and sleep, and not preceded by flashes of light or sound. She also had photophobia for two months. Generalized tonic-clonic seizures occurred once, 2 weeks prior to presentation, lasting about 5 minutes, aborted with an unascertained injection, and followed by postictal sleep. She is not a known epileptic, and there is no family history of seizures. She attained menarche at 11 years, and her neurodevelopment was normal.

The patient also complained of impaired vision mostly to far objects, palpitations, diaphoresis, and easy fatigability. There was no history of hypertension in the family.

Positive findings on examination were blood pressure (BP)—150/101 mmHg (right arm), 152/113 mmHg (left arm), 160/110 mmHg (left thigh), and 160/120 mmHg (right thigh) (readings were >99th centile for age, gender, and height centiles)). Her oxygen saturation was 97%. Weight (33 kg) and height (145.6 cm) measurements were below the 5^th^ centile for the age.

Brain computerized tomography (CT) scan revealed no abnormality. An electrocardiogram revealed features suggestive of left ventricular hypertrophy. Urinalysis and serum electrolytes, urea, and creatinine were within normal range.

**Figure 1:**
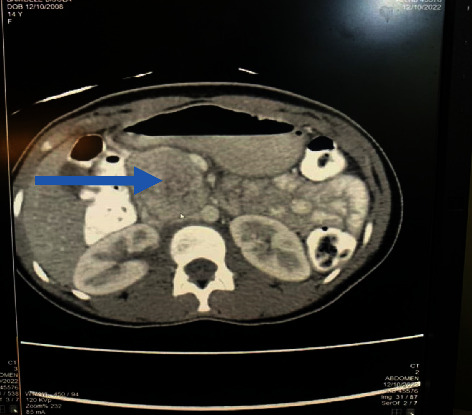
Contrast-enhanced axial computed tomography of the abdomen at the level of the kidneys showing an enhancing mass (arrow) just anterior and to the right of the aorta. It is compressing the inferior vena cava.

**Figure 2:**
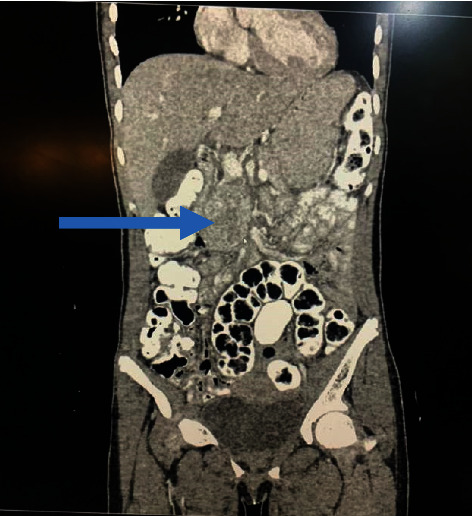
Contrast-enhanced coronal reformatted computed tomography of the abdomen. The mass (depicted by the blue arrow) is slightly to the right of the midline.

**Figure 3:**
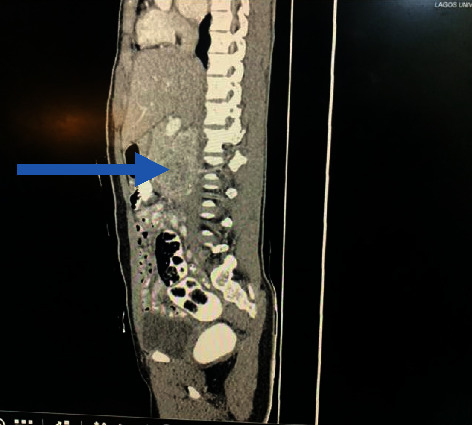
Contrast-enhanced sagittal reformatted computed tomography of the abdomen showing the posterior location of the mass (blue arrow).

A right para-aortic mass was seen on abdominal ultrasonography. The abdominal CT scan was suggestive of a paraganglioma (Figures 1–3). 24-hour urine normetanephrine was markedly elevated: 1695.34 mcg/24 h (100–500) while urine metanephrine was within the reference range: 105.11 mcg/24 h (50–250).

The multidisciplinary team involved in the preparation for surgery included the paediatric endocrinologists, anaesthesiologists, paediatric and cardiothoracic surgeons, radiologists, and the intensive care unit (ICU) team.

Intraoperative findings were a variegated right extra-adrenal mass adherent to the inferior vena cava and abutting on the abdominal aorta, firm to hard consistency, irregular lobes, and highly vascular, with thickened capsule. Histology results for the excised tumour showed nests and trabeculae of cells that were mildly pleomorphic with round to oval nuclei and salt and pepper nuclear chromatin pattern. The lesion was surrounded by a rim of fibrous tissue, and no capsular invasion, vascular invasion, or appreciable mitotic activity was seen. The report was in keeping with paraganglioma.

Intraoperative challenges were hypertension managed with sodium nitroprusside and labetalol while hypotension following tumour excision was corrected with noradrenaline infusion at 0.1 *μ*g/kg/min.

The patient was discharged home on the 10th postoperative day. Her blood pressure has remained normal (100 − 90/60 mmHg). Postoperative urine normetanephrines done a month after surgery had reverted to normal (192.39 *μ*g/24 hr) while repeat urine metanephrines remained normal at 117 *μ*g/24 hr. She is being followed up in the clinic and being planned for genetic studies, including family members when possible.

## 3. Discussion

Paragangliomas tend to be the commoner manifestation of chromaffin cell tumours in childhood [4, 7–9]. In a large Russian study of 520 patients with PCC/PGLs, 18% of children had extra-adrenal localization compared to 5.7% of adults, and this difference was statistically significant (*p* < 0.01) [9]. Similarly, a larger German study of 748 patients with PCC/PGLs seen over a 20-year period noted that children presented more frequently with extra-adrenal tumors (66.3% vs. 35.1%; *p* < 0.0001) in comparison with adults [8].

The average age at presentation of PCCs and PGLs in childhood is stated to be 11–13 years [2], and isolated case reports have also noted PGLs in adolescents [10] which is comparable to the age of the index patient. In relation to gender preponderance in PGLs, reports are varied, with either male or female preponderance or none [11–15].

Incidentally, all the 3 cases of PCC/PGL (index patient and 2 others with PCC) seen in our centre over a 6-year period happen to be females [15].

The clinical presentation of PGLs is variable. Those who secrete large amounts of catecholamines may cause signs and symptoms identical to those in patients with hyperfunctioning adrenal PCC. These include hypertension in the majority of cases, headaches, palpitations, and diaphoresis among others [2, 16, 17]. Anxiety, weight loss, and impaired vision have also been described. These clinical features were also observed in the index case. Sustained hypertension has been reported in 60–90% of childhood cases of PCC/PGLs in contrast to paroxysmal hypertension observed in about 50% of adult cases [2, 9].

An unusual presentation of PGL is seizures as occurred in the index patient prompting the first evaluation by a neurologist. Other reported cases of seizures as presentation of PCC/PGL or resolution after resection of the tumours are presented in Table 1.

Acute crises that occur in PCC/PGLs are probably due to a sudden massive release of catecholamines by the tumour. Even though cardiovascular events predominate, neurological symptoms such as seizures may dominate the clinical scenario [5]. Different mechanisms have been postulated as being responsible for seizures in patients with PCC/PGLs. These include hypertensive encephalopathy, reversible cerebral vasoconstriction syndrome (RCVS), or excitatory effects of noradrenaline on neurons [5, 6, 22].

Though the level of noradrenaline was not measured in the index patient, increased levels of normetanephrines (a metabolite of noradrenaline) were documented. Elevated catecholamines are thought to play a central role in the process of posterior reversible encephalopathy syndrome (PRES). This is a clinical syndrome of headache, visual disturbance, seizures, and confusion caused by the transient cerebral vasogenic edema occurring predominantly in the posterior circulation [5, 6]. As first described in patients with PCC/PGLs [22], PRES-related symptoms such as seizures, headaches, visual symptoms, confusion, or coma occur. Seizures can occur at onset or develop later in the process of PRES and are commonly generalized tonic-clonic in nature as seen in the index patient. Focal seizures can also occur [6].

Another possibility is RCVS. This syndrome was first described in a patient with PCC [23].

It is characterised by thunder-clap headaches and neurological symptoms though rare [6, 24]. Conditions of sympathomimetic overactivity as can occur in secretory PGLs are thought to be associated with vasoconstriction seen in the syndrome [25, 26].

Other reports have suggested that since norepinephrine plays a powerful activating role on neurons, increased activity of neurons causing seizures is noted in patients with PGL where secretion of norepinephrine predominates. It is noteworthy that some studies have also described the anticonvulsant effects of norepinephrine [6]. However, as Fitzgerald [27] proposed in his review in 2010 after examining hundreds of studies, there is a chance that NE plays its anticonvulsant property at an appropriate concentration but has a proconvulsant effect in either too high or too low concentrations [27]. The author [27] also noted that the final conclusions of the various studies depended on factors such as animal species, the strain, the model of epilepsy employed, and the location of the receptor.

Currently, the measurement of fractionated plasma and/or urine metanephrines (metanephrines and normetanephrines), which are highly sensitive tests (approaching 100% sensitivity), is recommended for the diagnosis of PCCs/PGLs. A greater than 4-fold rise over the reference range is associated with almost 100% probability [1]. Studies and case reports have noted that the 24-hour urine plasma normetanephrines or norepinephrine concentrations were usually higher in PGLs than in PCCs while metanephrines or epinephrine concentrations were mainly elevated in PCCs, but not in PGLs [10, 12]. This pattern was also noticed in the index patient. A proffered reason for the difference in secretion pattern is the reduced expression of phenylethanolamine N-methyltransferase (PNMT), the enzyme that converts norepinephrine to epinephrine in PGLs [1].

After confirming a PGL biochemically, imaging studies are useful in localizing the tumour which will also aid in surgery. The imaging characteristics of magnetic resonant imaging (MRI) scanning are quite typical for paraganglioma and preferred to computerized tomography (CT) scan. However, where the availability and affordability of MRI is limited as in our resource-constrained settings, CT scan still proves useful especially because of its outstanding spatial resolution [3]. In the study by Park et al. [12], all the tumours were visualised on CT.

The treatment of choice for paraganglioma is surgical resection [5]. Medical preoperative preparation is important to prevent and minimize dangerous complications from massive surges of released catecholamines from the tumour during surgery [3, 28, 29]. Intraoperative complications may include haemodynamic instability which may manifest as hypertension before tumour removal and hypotension after tumour isolation [29].

A limitation of this report is the inability of genetic testing of the patient and the family members because childhood PCC/PGLs, particularly PGL which the index patient had, is associated with a high risk of mutations in one of the PCC/PGL susceptibility genes. Additionally, angiography could not be done to confirm the RCVS.

## 4. Conclusion

Though endocrine causes of childhood hypertension are not common, they must be considered when evaluating aetiology of hypertension. Paragangliomas, which can be a cause, may present in an atypical pattern with seizures. Clinicians must strive to unravel the cause of seizures in a patient and not just manage with antiepileptic drugs. Measurement of BP in children is advocated as part of routine health care.

## Figures and Tables

**Table 1:** Case reports of PCC/PGL with seizures in children and young adults.

Authors/year	Age (years)	Gender	Presenting symptoms	Initial working diagnosis	Investigations, management, and outcome
Leiba et al. /2003 [18]	20	Male	Generalized seizures and coma	Focal seizures with secondary generalisation	Catecholamines and imaging confirmed the diagnosis of PCC. Had surgery with the resolution of seizures (1-year follow-up)
Wall et al. /2009 [19]	8	Female	Slow-growing cervical mass and progressive epilepsy	Grand mal epilepsy	Surgical resection of mass with histology confirming paraganglioma. Normal urine catecholamine levels. Resolution of epilepsy (13-year follow-up)
Chartan et al. /2011 [20]	4	Male	Headaches and status epilepticus	Not stated	Catecholamines and imaging confirmed diagnosis of PCC, had surgery, and was discharged. No complications or recurrent hypertension
Anderson et al. /2012 [5]	15	Female	Severe headache, vomiting, dizziness, blurred vision, and a tonic-clonic seizure	Not stated	Brain imaging showed haemorrhage, plasma noradrenaline was high, and MRI revealed a right adrenal PCC. Symptoms resolved and blood pressure normalised after resection of the tumour
Jung et al. /2012 [21]	15	Male	Hypertension, cyclic headache, and vomiting for 10 years. Seizure 6 weeks before the surgery	Not stated	Subcortical intracranial haemorrhage in the left frontal area. CT showed 2 masses in the left adrenal gland. Spent 1 day in ICU and discharged 10 days postsurgery with no noticeable complications and events

## Additional Points

*Take Home Messages*. (i) Paraganglioma is a rare cause of secondary hypertension in children. (ii) Atypical presentation can occur in the form of neurological complications such as seizures. (iii) A high index of suspicion for endocrine causes should be maintained when evaluating children with seizures and hypertension.
